# A cohort study of membranous nephropathy, primary or secondary

**DOI:** 10.1186/s12882-021-02338-6

**Published:** 2021-04-19

**Authors:** Maryam Arghiani, Boshra Hasan Zamani, Fatemeh Nazemian, Sara Samadi, Malihe Saber Afsharian, Mahmoud Habibzadeh, Saeid Eslami, Mahin Ghorban Sabbagh

**Affiliations:** 1 Kidney Transplantation Complications Research Center, Mashhad University of Medical Sciences, Mashhad, Iran; 2 Student research committee, Mashhad University of Medical Sciences, Mashhad, Iran; 3 Islamic Azad University, Mashhad Branch, Mashhad, Iran; 4 Razi pathology and genetic lab, Rasht, Iran; 5 Department of medical informatics, Faculty of Medicine, Mashhad University of Medical Sciences, Mashhad, Iran

**Keywords:** Membranous nephropathy, Phospholipase A2 receptor, PLA2R, IgG4, Primary MN, Secondary MN

## Abstract

**Background:**

Although IgG4 deposit against phospholipase A2 receptor (anti-PLA2R) is predominantly presented in the renal biopsy of patients with primary membranous nephropathy (MN), its diagnostic value of this immune complex has not been fully established.

**Methods:**

In this cohort study, 108 biopsy-proven MN patients with proteinuria were evaluated during two years follow up and were divided into primary and secondary groups. Renal biopsy specimens were pathologically assessed for IgG4 and PLA2R depositions by immunohistochemistry (IHC). Therefore, the relationships between staining severity, MN type and total proteinuria in all patients were determined.

**Results:**

Of 108 patients, 73.1% had primary MN and 26.9% were diagnosed as secondary form. IHC staining in patients with primary MN was positive for PLA2R in 76 (96.2%) and IgG4 in 68 (86.1%). Cases with positive PLA2R expression had a significantly higher rate among patients with mild to moderate stages (*P* = 0.03). No significant relationship was found between intensity of PLA2R and IgG4 deposits with proteinuria and serum creatinine. Based on our data, double positivity/negativity of PLA2R and IgG4 expression adds prominent information to the clinical data and were found to be useful and robust biomarkers for detection of primary MN patients with high sensitivity and specificity (97.1 and 96.3% respectively, PPV = 98.5% and NPV = 92.9%).

**Conclusions:**

Simultaneously expression of PLA2R and IgG4 in renal biopsy specimens of patients with MN could possibly be used as a potential diagnostic method to distinguish primary from secondary MN and also pathological severity of the disease.

**Supplementary Information:**

The online version contains supplementary material available at 10.1186/s12882-021-02338-6.

## Introduction

Membranous nephropathy (MN) is considered as an autoantibody-mediated glomerular disease, leading one of the causes of nephrotic syndrome among non-diabetic adults. The cause of most cases of MN is unknown and idiopathic [1].

MN refers to the early histological changes including thickening of the glomerular base membrane as a result of anti-podocyte targeted antibodies (IgG) depositions on the subepithelial region of the glomerular wall which may be accompanied by slight cell proliferation or infiltration. Patients with MN usually show manifestations such as severe proteinuria, edema, hypoalbuminemia and hyperlipidemia. Primary MN is identified as the most common type of the disease and secondary MN occurs by causative disease including autoimmune disease (most often systemic lupus erythematosus), infectious disease, drug reactions and malignancies served as approximately 25% of all types of MN [2]. Multiple studies have identified IgG antibodies against a podocyte-expressed protein namely M-type phospholipase A2 receptor type 1 (PLA2R) [3] that can be existed in the 75–85% of patients with primary MN [4, 5]. PLA2R has been identified as a main autoantigen on podocytes of majority of primary MN patients, while the immune complex involve in secondary MN is not pertinent to renal antigens [6, 7]. As demonstrated by clinical evidences, IgG4 subclass participate in autoimmune settings of primary MN. However, in secondary MN, the major antibodies are related to other subclasses of IgG [8]. Clinical studies support podocyte damage and consequent proteinuria caused by accumulation of subepithelial autoantibodies deposition. PLA2R describes as a mannose receptor in human [9] and consider being highly produced through podocytes, alveolar type II epithelial cells and also neutrophils, so that autoantibodies against PLA2R uniquely cause nephrotic syndrome with no serious damage to other organs [10, 11]. Using immunohistochemistry (IHC) and/or immunofluorescence (IF), IgG4 antibodies against anti-PLA2R can be identified in primary MN patients. The PLA2R antigen less commonly found on the podocytes in normal kidneys or another cases of glomerular disease [12]. Overall, a robust relationship between PLA2R staining and anti-PLA2R circulating antibodies can specially be found [13, 14] at the time of autoantibody measurements during biopsy evaluations [15]. Based on previous studies using delayed serum sampling, evaluation of PLA2R antigen in biopsy samples can be more sensitive rather than serological assessment in order to detection of PLA2R-associated MN [15]. Since antibodies against PLA2R are detectable in nearly 70–80% of adult MN patients with no secondary reasons, the clinical significance of anti- PLA2R can be taken into account as a biomarker of primary MN detection [3, 16]. Although many clinical studies have investigated the role of serum PLA2R antibodies among MN patients [17–19], limited data has been provided in terms of simultaneously staining of PLA2R and IgG4 in renal tissues of MN patients [20], particularly in large samples. To our knowledge, the glomerular deposition of both PLA2R and IgG4 in primary and secondary forms of membranous nephropathy has not previously been evaluated in large samples of Iranian population. The aim of the current study was to evaluate the potential value of immunohistochemical staining for differential detection of primary and secondary MN and pathological severity of the disease.

## Materials and methods

### Study design and patients

**Fig. 1:**
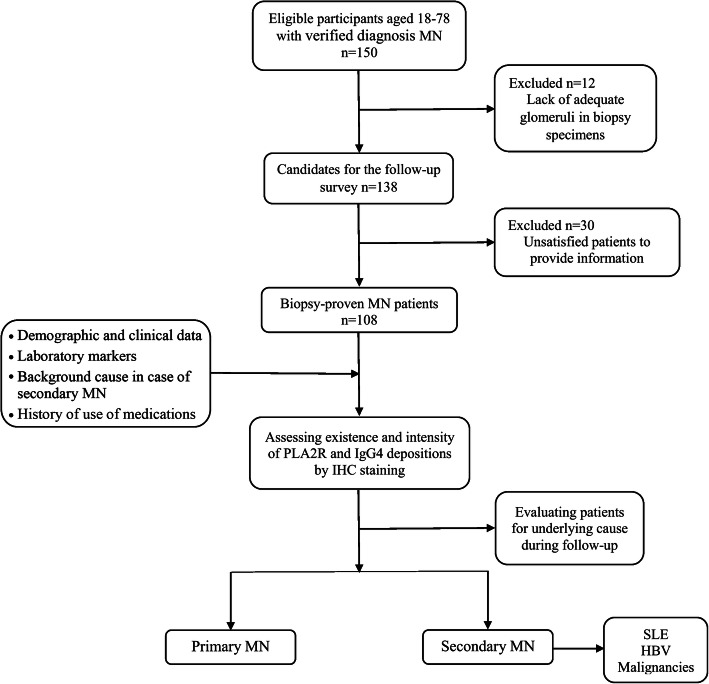
Flowchart of the cohort

This cohort study performed during 2017–2019 in north east of Iran. One hundred-fifty eligible individuals participated which out of these, 108 biopsy-proven MN patients recruited in our study. Eligible participants 18 to 78 years old with verified diagnosis of MN were recruited. Eligibility was determined based on clinical presentations according to nephrotic syndrome and definitive diagnosis of MN using pathological studies of renal biopsy specimens, in addition to obtaining consent to assess patient documents and pathological samples taken from their kidney biopsy. Nevertheless, patients with lack of adequate glomeruli in biopsy specimens for IHC staining (*n* = 12) as well as unsatisfied patients to provide information (*n* = 30) were excluded from our study. Assessments included determination of demographic (age and sex), clinical data (such as medical history, course of the illness and clinical presentations), laboratory markers (including 24-h urine protein measurement, serum creatinine assessment), type and stage of the disease, background cause in case of secondary MN, as well as medication history. All samples were examined for the existence and intensity of PLA2R (Medaysis, California, USA) and IgG4 (Biocare Medical, California, USA) depositions by IHC staining under similar conditions, with the same kits and by a pathoimmunologist. Participants were underwent a close monitoring and separated into two categories of primary and secondary MN, according to the presence or absence of underlying cause. Patients diagnosed as primary MN at the beginning of study were transferred to the secondary form of MN while identifying an underlying cause during follow-up years based on clinical and experimental evidences (Fig. 1).

The current study was approved by Ethics Committee of Mashhad University of Medical Sciences and informed written consent was also taken from all enrolled members. New samples were also prepared from biopsy specimens of each patient to assess IHC staining in case of PLA2R and IgG4 markers. Samples were examined for the existence of PLA2R and IgG4 depositions by IHC staining as well as the amount of the deposits in the specimens.

### Identifying MN patients with various causes of disease

The detection of MN was occurred based on pathological assessments such as immunofluorescence and light microscopy of renal biopsy samples. Idiopathic MN was diagnosed followed by elimination of secondary causes including: 1) infection with hepatitis B, 2) malignancies like prostate, Gastrointestinal (GI) cancer and chronic lymphocytic leukemia (CLL) or lymphoma, 3) autoimmune diseases such as systemic lupus erythematosus (SLE), as well as 4) drugs include as penicillamine and nonsteroidal anti-inflammatory drugs [6, 21]. Microscopic findings such as mesenchymal hypercellularity and endocapillary proliferation were identified as secondary MN pathology. Immunofluorescent microscopic findings of C_1_q, IgM and IgA depositions, glomeruli full house view and IgG deposits in tubular basement membrane were all in favor of secondary MN, in particular SLE. During a 24- month period, patients were followed up periodically by clinical and laboratory examinations and transferred to the secondary group if evidence of an underlying cause emerged.

### IHC assay for PLA2R and IgG4 identification in glomerular deposits

IHC studies were also examined in terms of IgG4 and PLA2R evaluation. In addition, patient samples were analyzed based on pathological features, so that respective pathologic stage was determined for each patient with MN. Moreover, the association between the amount of deposits and type of the disease and also severity of proteinuria was evaluated in both conditions of totally and in various sub-groups of patients. SLE patients were diagnosed based on particular criteria released in 2012 from the Systemic Lupus International Collaborating Clinics (SLICC) and membranous lupus nephritis were identified regarding to the factors revised by the International Society of Nephrology and Renal Pathology Society (ISN/RPS) in 2003 [22, 23]. Hepatitis B virus (HBV) infection was identified through detection of serological markers [24]. Following finding criteria in case of malignancy related MN detection, it is considered as malignancy at the time of kidney biopsy and/or is detected during two years follow-up, and when there is no other secondary cause of MN [25].

### Statistical analysis

All statistical analyses were performed using SPSS software, version 22. Results obtained from normal data were reported as mean ± SD. Baseline characteristics of participants with primary and secondary MN were compared by independent two-sample t-test for normal distributed parameters, chi-square for categorical data, and Fishers exact test. Significance was considered as *P* values less than 0.05.

## Results

### Clinical characteristics

In the present study, 108 patients suffering from MN with mean age of 44.21 ± 13.32 were enrolled. Out of these, 79 patients (73.1%) had primary MN, while 29 patients (26.9%) were diagnosed as secondary MN. The underlying causes of the secondary MN were recognized which most common of them were related to the autoimmune disease (72.4%). The main secondary causes in patients with secondary MN were included as 72.4% SLE (21 patients), 13.8% HBV (4 patients) as well as 13.8% with malignancies (4 patients consist of a prostate cancer, a GI cancer, a lymphoma and a case of CLL). Pathologic studies for MN patients staging indicated that 9 patients (8.3%) categorized in the severe stage, most of patients showed moderate disease (80 patients, 74.1%) and the rest of them (19 individuals, 17.6%) possessed mild stage. Overall, 61.1% of participants were men, as women accounted for 38.9% of studied population. Clinical characteristics of studied population (*n* = 108) have been shown in Table 1. According to these findings, 10.2% of participants were diabetic and 63% had hypertension. Moreover, 84.3% took angiotensin converting enzyme inhibitor (ACEi) or angiotensin receptor blocker (ARB) drugs and the most common immunosuppressive regimen was prednisolone plus calcinurin inhibitor (59.2%).

**Table 1:** Clinical characteristics of studied population (*n* = 108)

	Parameter	*p* value
	Age (years)	44.21±13.32
	Gender (male)	66 (61.1%)
	Diabetes	11 (10.2%)
	HTN	68 (63%)
Immunosuppressive treatment	ACEi/ARB administration	91(84.3%)
	PDN + CNI	61 (59.2%)
	PDN + CNI + MMF	9 (8.7%)
	Rituximab	2 (1.9%)
	Ponticelli regimen	10 (9.7%)
	PDN	7 (6.8%)
	None	14 (13.6%)

*Abbreviations*: *HTN* hypertension, *ACEi/ARB* angiotensin-converting enzyme inhibitor/angiotensin II receptor blocker, *PDN* prednisolone, *CNI* calcineurin inhibitor, *MMF* mycophenolate mofetil

Table 2 illustrates the distribution rate of diabetes, hypertension, administration of ACEi and ARB drugs, treatment regimen type for suppressing immune system and disease stage among patients with primary and secondary MN. Regarding to the results, disease stage showed significant difference between two groups of primary and secondary MN (*p* = 0.014). Data showed that mean value for 24-h urine protein and serum creatinine were 5.09 ± 2.64 g/day and 1.30 ± 1.23 mg/dl respectively. However, results from 24-h urine protein and serum creatinine showed no significant difference between primary and secondary MN (*p* = 0.8 and *p* = 0.4 respectively).

**Table 2:** Baseline features of patients across primary and secondary MN

		Primary MN	Secondary MN	*p* value
Disease stage	Mild	16 (20.3%)	3 (10.3%)	
	Moderate	60 (75.9%)	20 (69%)	0.014
	Severe	3 (3.8%)	6 (20.7%)	
Gender	Male	50 (63.3%)	16 (55.2%)	0.44
HTN	Positive	51 ((64.6%)	17 (58.6%)	0.57
Diabetes	Positive	9 (11.4%)	2 (6.9%)	0.49
ACEi/ARB administration	Positive	68 (86.1%)	23 (79.3%)	0.39
Immunosuppressive treatment	PDN + CNI	48 (60.8%)	13 (44.8%)	
	PDN + CNI + MMF	4 (5.1%)	5 (17.2%)	
	Rituximab	2 (2.5%)	-	
	Ponticelli regimen	8 (10.1%)	2 (6.9%)	
	PDN	5 (6.3%)	2 (6.9%)	
	None	9 (11.4%)	5 (17.2%)	

*Abbreviations*: *MN* membranous nephropathy, *HTN* hypertension, *ACEi/ARB* angiotensin-converting enzyme inhibitor/angiotensin II receptor blocker, *PDN* prednisolone, *CNI* calcineurin inhibitor, *MMF* mycophenolate mofetil

### IHC studies for PLA2R detection in glomerular deposits

**Fig. 2:**
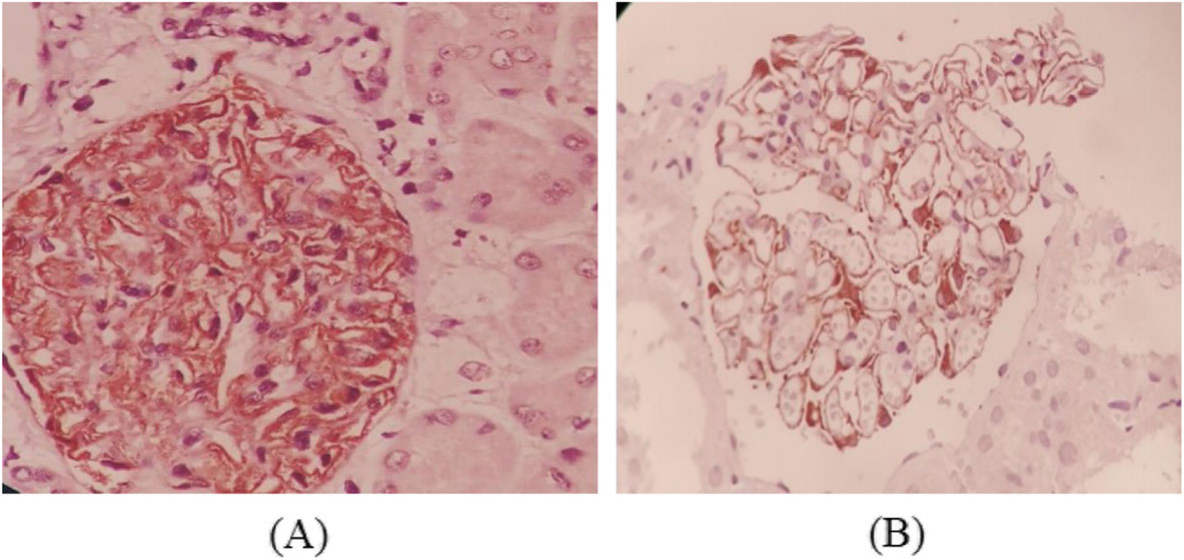
Immunohistochemical staining (× 400). **a** PLA2R deposition and **b** Glomerular IgG4 deposits in a precisely granular pattern in sub-epithelial deposition of glomerular capillary wall

IHC investigations indicated PLA2R deposition in a precisely granular pattern in sub-epithelial deposition located in glomerular capillary wall (Fig. 2, a).

Based on our findings, 96.2% of patients with primary MN indicated positive glomerular PLA2R expression, while only 3.5% (one patient) of those who had secondary MN showed PLA2R deposition. This one patient among 29 secondary MN was related to the patient of CLL which consist 25% of malignancy group. Among 76 primary MN patients who had PLA2R in glomerular deposits, the intensity of the staining for 47 patients were high (61.8%), and 28 patients were moderate (36.8%). IHC staining for those cases with very mild positivity were identified as negative findings in the present study (Table 3). Furthermore, the sensitivity and specificity of PLA2R positive expression in differentiating primary MN from secondary ones were 96.2 and 96.6% respectively. Likewise, positive predictive value (PPV) was found to be 98.7%, while negative predictive value (NPV) was 90.3%.

**Table 3:** The prevalence of PLA2R, IgG4 and staining intensity assessed by IHC in MN patients

		Primary MN (*n* = 79)	Secondary MN (*n* = 29)	*p* value
Glomerular PLA2R Ag expression	Positive	76 (96.2%)	1 (3.5%)	<0.001
	Negative	3 (3.8%)	28 (96.5%)	
Staining intensity^a^	Moderate	29 (38.2%)	-	-
	severe	47 (61.8%)	1 (100%)	
Glomerular IgG4 expression	Positive	68 (86.1%)	3 (10.3%)	<0.001
	Negative	11 (13.9%)	26 (89.7%)	

*Abbreviations*: *MN* membranous nephropathy, *PLA2R* phospholipase A2 receptor, *IgG4* Immunoglobulin G4

^a^IHC staining for those cases with very weak positivity were considered as negative

### IgG4 in glomerular deposits

**Fig. 3:**
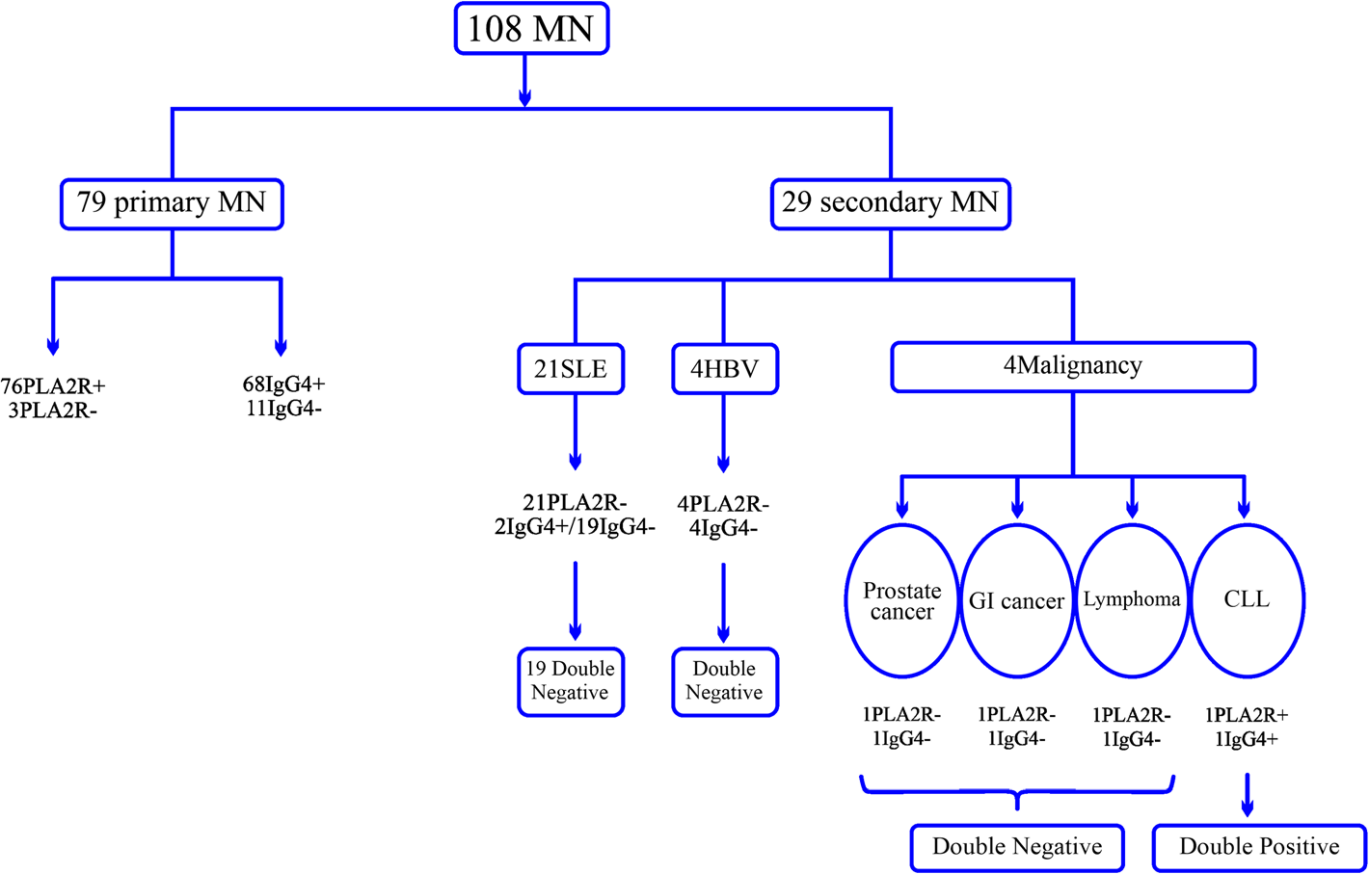
Diagram of PLA2R and IgG4 expression among primary and secondary MN

In 68 of 79 patients with primary MN (86.1%), glomerular IgG4 deposits were observed (Fig. 2, b). However, IgG4 deposits among secondary MN was positive for three patients (10.3%) (Table 3). These three IgG4 positive cases of MN with secondary causes were comprised of two SLE patients in addition to one patient with malignancy (Fig. 3). In order to diagnosing primary MN from secondary forms, sensitivity of IgG4 positive expression was 86.1% and specificity was 89.7%. PPV and NPV related values were reported as 95.8 and 70.3% respectively.

### The relationship between PLA2R and IgG4 in glomerular deposits

As data indicated by IHC staining, the positivity rates of glomerular PLA2R and IgG4 expression were significantly higher in primary MN patients rather than those with secondary MN (*p* < 0.0001). In majority of patients with various causes of secondary MN, staining was negative for PLA2R and IgG4. Among 79 primary MN patients, 68 patients possessed both PLA2R and IgG4 deposits together and eight patients expressed PLA2R but no deposition for IgG4. Moreover, PLA2R and IgG4, were found to be double negative in two patients with primary MN. Out of 4 patients with malignancies within secondary MN patients, only a case of CLL was double positive for PLA2R and IgG4 deposition (25%). Of four patients with HBV, no one exhibited positive glomerular PLA2R and IgG4 expression. In addition, 19/21 SLE patients showed double negative pattern for PLA2R and IgG4 deposition (91.5%) (Fig. 3). The sensitivity and specificity of double positivity/negativity of PLA2R and IgG4 expression in case of diagnosing primary MN from secondary forms were found to be 97.1 and 96.3% respectively. We also examined the positivity of IHC staining between different pathologic stages of patients for markers of PLA2R and IgG4. As results indicated, a significant difference have been found among patients with various types of disease severity according to their pathologic stage in PLA2R staining (*p* = 0.03) (Table 4).

**Table 4:** Expression of PLA2R and IgG4 in different stages of MN according to pathologic stage

		Mild (*n* = 19) (stage I)	Moderate (*n* = 80) (stage II)	Severe (*n* = 9) (stage III-IV)	*p* value
PLA2R	Positive	14 (73.7%)	60 (75%)	3 (33.3%)	0.03
	Negative	5 (26.3%)	20 (20%)	6 (67.7%)	
Staining intensity	Moderate	6 (42.9%)	23 (38.3%)	-	0.37
	severe	8 (57.1%)	37 (61.7%)	3 (100%)	
IgG4	Positive	12 (63.2%)	56 (70%)	3 (33.3%)	0.08
	Negative	7 (36.8%)	24 (24%)	6 (67.7%)	

*Abbreviations*: *MN* membranous nephropathy, *PLA2R* phospholipase A2 receptor, *IgG4* Immunoglobulin G4

Moreover, the positivity rate of staining in patients presenting mild and moderate stage of the disease have been found to be significantly higher in patients with severe stage of the disease. Nevertheless, in IgG4 staining, there was no significant difference in case of various staining intensities regarding to the pathologic stages.

### Association of PLA2R and IgG4 deposits with clinical features

The association of proteinuria and serum creatinine with PLA2R and IgG4 staining intensity was also evaluated. According to our findings, no significant relationship was found between intensity of PLA2R and IgG4 deposits with proteinuria and serum creatinine.

## Discussion

According to our data, the most cases of the patients were related to the primary MN. Autoimmune disease was the most common cause among secondary MN patients. Based on pathological evidences, 8.3% of cases were severe (stage III-IV), 74.1% moderate (stage II) and 17.6% showed mild (stage I) MN. Our results indicated that the rate of severe stages of the disease was significantly higher in patients with secondary MN. Using IHC staining, two markers of PLA2R and IgG4 in glomerular deposition of MN patients were evaluated to examine the potential value of the assessment for differential detection of primary and secondary MN. According to our findings, high sensitivity and specificity of PLA2R and IgG4 expression were found in diagnosing patients with primary and secondary MN, suggesting PLA2R and IgG4 expression as potential and robust biomarkers for detection of primary MN patients. Among patients with primary MN that were positive for PLA2R expression, the IHC staining intensity in 61.8% of cases was high, while 36.8% of patients exhibit moderate receptor expression. The positivity rate for PLA2R and IgG4 were significantly higher in primary MN rather than secondary forms. Double positivity (PLA2R+/IgG4+) was presented in 68 cases (86.07%) with primary MN, whereas patients with secondary MN were double negative (89.65%) for both markers of PLA2R and IgG except for three cases (3/29). Regarding to the disease pathologic stage, the distribution of PLA2R positive cases in patients with mild and moderate degree of primary MN was significantly higher than those with severe disease. However, our results showed no significant difference between these pathologic stages and IgG4 expression. Likewise, no significant association was found between various expression intensity of PLA2R and different disease stages according to the pathological view. As indicated by Yeo et al., primary MN can be distinguished from secondary forms through immunohistochemical detection of PLA2R and IgG4 deposits from renal biopsy with acceptable sensitivity and specificity of the test. Simultaneous positivity rates for PLA2R and IgG4 demonstrated an increased specificity in case of primary MN detection [26]. IHC staining has previously been shown to be the most sensitive technique, identifying MN patients correlated with anti-PLA2R antibodies [27]. These evidences were in accordance with our results, supporting diagnostic value of these markers in discriminating primary from secondary MN patients. Similarly, no significant relationship was found between primary and secondary MN in terms of proteinuria and creatinine. A meta-analysis recruited 28 studies was conducted to explore the accuracy of diagnostic value of anti-PLA2R and glomerular PLA2R antigen and found them as useful discriminating tools for differentiation of idiopathic MN from non-idiopathic form [28]. Results of receiver operating characteristic (ROC) curve analysis obtained from 64 MN patients showed that combined identification of serum anti-PLA2R antibodies, glomerular PLA2R, and the IgG deposition led to achieve higher sensitivity of detection, indicating its prominent role in higher detection efficacy of idiopathic MN [29]. In consistent with our study, an increased rate of positivity of glomerular PLA2R expression mainly IgG4 deposit was observed among patients with primary MN. Our data revealed that double positivity/negativity of PLA2R and IgG4 expression are considered potential biomarkers for diagnosing primary MN patients with high sensitivity (97.1%) and specificity (96.3%), representing PPV and NPV of 98.5 and 92.9% respectively. Although simultaneous determination of PLA2R and IgG4 expression provide enhanced sensitivity and specificity in terms of discriminating primary MN from secondary form, the experiment costs may also increase.

Data from 95 MN patients underwent IHC assessment against three indicators of PLA2R, IgG4 and THSD7A have revealed a classification of specimens into double-positive (PLA2R+/IgG4+) and triple-negative (PLA2R−/IgG4−/THSD7A−), significantly lowering the rate of false-negative or false-positive data and supporting the useful application of MN clinical characterization [30]. Moreover, indirect immunofluorescence test was applied to examine Anti-PLA2R autoantibodies in a large study include 252 idiopathic MN samples and 184 pathological controls such as secondary MN. The result of this study confirmed that the positivity of anti-PLA2R among MN patients does not necessarily rule out a secondary cause for MN patients, particularly within those who have risk markers of malignancies. Therefore, the cause of the link between tumor and MN related different diseases and also probably underlying pathways of autoimmunity-induced malignancies remains unknown [31]. Interestingly, our study illuminated that the IHC staining in patients with secondary MN was double positive for PLA2R and IgG4 in a case of cancer (1/29) and the highest staining rate was dedicated to malignancies, suggesting the similarity between these receptors with antigens relevant to the malignancies. Since all of cases were not followed for two years in our study and a few cases had nine months follow-up, this can be considered as a limitation of our study. In addition, because this was a historical cohort, we did not have adequate samples to do further experiments such as new MN antigens including THSD7a. Moreover, the main focus of the current study was to evaluate the diagnostic value of PLA2R and IgG4 combination on primary and secondary membranous nephropathy in a population-based cohort.

Dual positivity of anti-PLA2R antibodies and PLA2R antigen has been found as markers of enhanced disease activity and reduced chance of remission, in comparison with those of just positive for PLA2R antigen. Similarly, Bi-negativity to both parameters associated with secondary MN [32, 33]. Based on our results, IHC determination of PLA2R and IgG4 can be associated with improved diagnostic accuracy of primary MN. This is in line with other clinical studies which opined that high expression of IgG4 in patients with idiopathic MN occur in addition to increased expression of PLA2R in renal samples of these patients and may lead to enhance differential power of detection primary and secondary MN [20, 26, 34].

## Conclusions

Using data from the historical cohort, identifying double positive expression of PLA2R and IgG4 plays a prominent role in discriminating diagnosis of idiopathic MN from secondary cases. Nevertheless, as malignancies and autoimmune diseases may be positive for these antibodies, differential diagnosis of primary MN from secondary form requires exclusion of these cases.

## Supplementary Information

**Additional file 1: Table S1**. Data from 24-hour urine protein and serum creatinine showed no significant difference between primary and secondary MN.

## Data Availability

The datasets used and/or analyzed during the current study are available from the corresponding author on reasonable request.

## Abbreviations

PLA2R: Phospholipase A2 receptor
MN: Membranous nephropathy
IHC: Immunohistochemistry
IF: Immunofluorescence
GI: Gastrointestinal
CLL: Chronic lymphocytic leukemia
SLE: Systemic lupus erythematosus
SLICC: Systemic Lupus International Collaborating Clinics
ISN/RPS: Nephrology and Renal Pathology Society
HBV: Hepatitis B virus
ACEi: Angiotensin converting enzyme inhibitor
ARB: Angiotensin receptor blocker
PPV: Positive predictive value
NPV: Negative predictive value
ROC: Receiver operating characteristic
