# Supplementary material for: A cohort study of membranous nephropathy, primary or secondary
Source: BMC Nephrol. 2021 Apr 19;22:138. doi: 10.1186/s12882-021-02338-6 (PMC8056567; doi:10.1186/s12882-021-02338-6)
Supplement: Supplementary file 1 — Additional file 1: Table S1. Data from 24-hour urine protein and serum creatinine showed no significant difference between primary and secondary MN. [file 12882_2021_2338_MOESM1_ESM.docx]

Supplementary table:

| Table S1. Clinical features across primary and secondary MN. | | | |
| --- | --- | --- | --- |
|  | Primary MN (n=79) | Secondary MN (n=29) | p value |
| Age (year) | 44.28±12.75 | 44.03±15.01 | 0.93 |
| SCr (mg/dl) | 1.21±0.49 | 2.25±1.56 | 0.4 |
| U-pro (g/day) | 5.12±2.69 | 5.02±2.5 | 0.86 |
| Abbreviations: MN, membranous nephropathy; SCr, serum creatinine; U-pro, 24-hour urine protein  Values are presented as mean ±standard deviation in Primary MN and Secondary MN  SCr, serum creatinine; U-pro, 24-hour urine protein | | | |
